# Attention-Based Deep Entropy Active Learning Using Lexical Algorithm for Mental Health Treatment

**DOI:** 10.3389/fpsyg.2021.642347

**Published:** 2021-03-30

**Authors:** Usman Ahmed, Suresh Kumar Mukhiya, Gautam Srivastava, Yngve Lamo, Jerry Chun-Wei Lin

**Affiliations:** ^1^Electrical Engineering and Mathematical Sciences, Western Norway University of Applied Sciences, Bergen, Norway; ^2^Department of Mathematics and Computer Science, Brandon University, Brandon, MB, Canada; ^3^Research Centre for Interneural Computing, China Medical University, Taichung, Taiwan

**Keywords:** adaptive treatments, internet-delivered interventions, NLP, text clustering, word sense identification

## Abstract

With the increasing prevalence of Internet usage, Internet-Delivered Psychological Treatment (IDPT) has become a valuable tool to develop improved treatments of mental disorders. IDPT becomes complicated and labor intensive because of overlapping emotion in mental health. To create a usable learning application for IDPT requires diverse labeled datasets containing an adequate set of linguistic properties to extract word representations and segmentations of emotions. In medical applications, it is challenging to successfully refine such datasets since emotion-aware labeling is time consuming. Other known issues include vocabulary sizes per class, data source, method of creation, and baseline for the human performance level. This paper focuses on the application of personalized mental health interventions using Natural Language Processing (NLP) and attention-based in-depth entropy active learning. The objective of this research is to increase the trainable instances using a semantic clustering mechanism. For this purpose, we propose a method based on synonym expansion by semantic vectors. Semantic vectors based on semantic information derived from the context in which it appears are clustered. The resulting similarity metrics help to select the subset of unlabeled text by using semantic information. The proposed method separates unlabeled text and includes it in the next active learning mechanism cycle. Our method updates model training by using the new training points. The cycle continues until it reaches an optimal solution, and it converts all the unlabeled text into the training set. Our in-depth experimental results show that the synonym expansion semantic vectors help enhance training accuracy while not harming the results. The bidirectional Long Short-Term Memory (LSTM) architecture with an attention mechanism achieved 0.85 Receiver Operating Characteristic (ROC curve) on the blind test set. The learned embedding is then used to visualize the activated word's contribution to each symptom and find the psychiatrist's qualitative agreement. Our method improves the detection rate of depression symptoms from online forum text using the unlabeled forum texts.

## 1. Introduction

According to a new World Health Organization (WHO) survey, the COVID-19 pandemic has disrupted mental health services in 93% of countries worldwide^1^. In contrast, mental health demand has increased due to lockdown of affected areas as a prevention measure. Any lockdown results in increasing physiological stress factors that include fears of illness and uncertainty of the future (Troyer et al., 2020). Social isolation, lack of interactions during education, and/or work also causes emotional stress resulting in a generally worse state for public mental health. Front-line health workers also suffer from anxiety and depressive symptoms due to fear of illness, lack of protective equipment, social disconnection, and a high-stress environment. Depression instances have been shown to be high during lockdown (Karmen et al., 2015). Initially, it is a reaction to life that a person never imagined. Since there are many unknowns to what causes depression, various things are often connected to its research. Diverse information provided by vast and growing literature includes various reports on how to tackle depression. Extracting useful knowledge is still difficult because of these conflicting reports (Ebadi et al., 2020). A combination of recent events and longer-term and/or personal factors trigger depression rather than just a single immediate issue or event (Mukhiya et al., 2020a). Although everyone is different, identifying the cause or change in difficult circumstances cannot be possible always (Losada and Gamallo, 2018). The most important thing is to recognize the early signs and symptoms for depression and seek support at an early stage. Nowadays, numerous Internet forums and social media platforms enable individuals to contact each other and share their suffering, pain, and potential treatment options anonymously (Low et al., 2020). People worldwide can share their ideas and experiences without being exposed (Mühleck et al., 2019). Online detection can be a proactive and promising approach to distinguish high-risk people. It can encourage timely mediation and can help improve general well-being (Neuraz et al., 2020).

The WHO ranks *depression* as one of the world's most disabling diseases (James et al., 2018). It has become a common illness worldwide, with more than 264 million people affected (James et al., 2018). Depression that goes untreated may become more severe and cause lifelong suffering (Mazza et al., 2020). Depression, at its worst, can lead to suicide. WHO reported that close to 800, 000 people die due to suicide every year (James et al., 2018). Suicide is the second leading cause of death among 15–29-year olds. Between 76 and 85% of people in low- and middle-income countries receive no treatment for their disorder. Barriers to effective care include a lack of resources, lack of trained healthcare providers, inaccurate assessment, and social stigma associated with mental disorders (James et al., 2018). Social stigma, shyness, and anxiety about discussing the problem are the key barriers that keep patients reluctant to treatment. People often feel embarrassed, ashamed, and fear of having to undergo a probing examination of their psychological pain (Mukhiya et al., 2020b). For these reasons, they may not want to acknowledge that they are depressed or seek treatment.

The healthcare systems are facing a global challenge for preventing and treating mental health problems. The overburdened health system faces economic and technical pressure to develop an adaptive system that will reduce waiting time and provide intervention by reducing cost. Internet-delivered Psychological Treatment (IDPT) can help to overcome mental and physical distress for a large population and using fewer resources (Mukhiya et al., 2020b). Most of the existing solutions are tunnel-based, inflexible, and non-interoperable (Mukhiya et al., 2020c). Current models lack adaptive behavior, which in turn results in lower user adherence and more dropouts (Konrad et al., 2015). Treatments should be considerate of methods available for users to adopt the treatments. This user adoption can be achieved by using an IDPT system in a way that user behavior itself should be taken into consideration. This user behavior includes different preferences and needs according to their environment and mental health symptoms(Mukhiya et al., 2020b). In this study, we aim to extract depression symptoms from patient's authored text. We attempt to identify and visualize using the deep attention-based method. Mostly, a given patient expresses their mental health issues in their communication. Based on the patient's own words, we consider the extraction of the factors that result in depression-related symptoms. Using an online interactive tool (ICT) that provides contextual information and visualization for adequate mental health, we aim to assist in providing prevention measures.

This paper address how to extract depression symptoms in mental health interventions using Natural Language Processing (NLP) and attention-based in-depth entropy active learning. For this purpose, we propose the method based on synonym expansion by semantic vectors. We cluster the semantic vectors based on the semantic information derived from the context in which it appears. The resulting similarity metrics help to select the subset of unlabeled text by using semantic information. Our method separates unlabeled text and includes it in the next active learning mechanism cycle. Our method updates the model training by using the new training points. The cycle continues until it reaches the optimal solution, and it converts all the unlabeled text into the training set. The objective of this research is to increase the trainable instances using a semantic clustering mechanism. Our method helps to reduce data annotation tasks and helps in the generalization of the learning system. The proposed framework achieved 0.85 ROC that shows the synonym expansion semantic vectors help enhance training accuracy while not harming the results.

The rest of the paper is structured as follows. Section 2 outlines the related works. Section 3 outlines the main methodology used to set up the experiment, collect data, and build the model. Section 4 discusses the results and findings. Finally, section 5 concludes with summary and future works.

## 2. Related Work

Several efforts have been attempted to improve depression detection using computer-aided methodologies. This section provides an overview of approaches that have been proposed in this regard.

Fliege et al. (2005) proposed an Item Response Theory (IRT) based Computer Adaptive Tests (CAT) to measure depressive symptoms (Depression-CAT, D-CAT). They aimed to develop an application using real patient data that measure depressive symptoms severity and promise to enhance measurement precision and reduce respondent's burden. Progress in measurement was achieved by utilizing an adaptive questionnaire rather than a static questionnaire. The information from previously replied questions was utilized to select the next most suitable questions. Asking those most relevant questions for every individual patient's CAT made it conceivable to introduce fewer things and accomplish greater measurement precision over the whole range of a construct. However, some problems remained unresolved. Such as the effect of differences in item order was unknown. It was also unknown that the varying response options within one test influences response behavior.

Lehrman et al. (2012) proposed another technique focusing on automatic analysis of short written texts on the bases of relevant linguistic text features to distinguish whether the authors of such texts are suffering from distress. It performed NLP using supervised machine learning. This study essentially concentrates on some fundamental supervised classification methods and text-based features to automatically classify mental affect states in short texts based on just a small dataset. This technique exemplifies a binary classification problem, where short texts are classified as either distressed or non-distressed. Four text classes were at a more fine-grained level: high distress, low distress, response, and happy. Any post expressing an active intent to harm someone or oneself was classified as high distress, while posts are only discussing bad feelings were usually classified as low distress—the annotated dataset of short written texts for the work. A dataset consisting of 200 posts from various public online forums dealing with mental well-being was utilized. Machine learning algorithms such as Naive Bayes, Maximum Entropy, and Decision Tree were applied to this dataset. They report an accuracy of 54.5% vs. a baseline of 30.5% when classifying four ways based on the level of distress.

Dinakar et al. (2014) presented a stacked generalization modeling approach to analyze online community youngsters under stress. In the first place, they trained an ensemble of base models for predicting individual labels, namely a support vector machine with a linear kernel (SVM-L), a radial basis function kernel (SVM-R), and a stochastic gradient boosted decision trees (GBDT) models. These models are trained for text classification to categorize into 23 themes. The SVM-L, SVM-R, and GBDT for each code were combined into a meta-feature set fed into a meta-classifier. The meta-features are made up of the individual base classifier. Features for base classifiers included unigrams, bigrams, part-of-speech bigrams, and tf-idf filtered via chi-squared feature selection and additional hand-coding features. The base classifiers' output was the vector of predictions. The decision function scores for each prediction, two along with the topic distribution from the L-LDA model for a given story then became meta-features for the suite of meta-learners. They analyzed 7,147 personal stories shared by distressed teenagers on a popular teen-help website.

Choudhury et al. (2013) used the behavior of youth and Twitter users in general to detect any sign of depression. They aimed to build a machine learning based model that can detect and rely on several signs from social media behavior to predict the potential depression of some users at early stages. The authors developed a crowdsourcing solution to the problem of developing a ground truth dataset. Annotators were recruited from Amazon Mechanical Turk and required to take a Center for Epidemiologic Studies Depression Scale test. They were asked a series of questions regarding their history of depression and current depression status. The Mechanical Turkers who finished the questionnaire were requested for their Twitter user name, which was then used to pull their Twitter feed, resulting in a ground truth depressed/not depressed dataset. After that, a machine learning classifier was trained on the depressed/not-depressed data using features derived from both the tweet text and network features such as several followers. That classifier was applied to an extensive dataset of geolocated Twitter data from the United States, yielding a strong positive correlation with Centers for Disease Control depression statistics. Choudhury et al. (2013) presented a study of predicting depression from tweets by analyzing more than 2 million posts of 476 users. The best performance was acquired by SVM classifier with a set of behavioral features, for example, the occurrence of pronouns, use of swearing and depression terms, tweet replies, just as posting time, and frequency.

Another experimental study has analyzed mental health phenomena in publicly available Twitter data (Chen E. et al., 2020). They gathered data for a range of mental illnesses quickly and cheaply to identify various mental health disorder symptoms such as depression, bipolar disorder, and seasonal affective disorder. They conducted a Linguistic Inquiry Word Count (LIWC) analysis of each disorder to measure deviations in each illness group from a control group, replicating previous findings for depression and providing new findings for bipolar, PTSD (Post-traumatic stress disorder), and SAD (Social-Anxiety Disorder) (Chen E. et al., 2020; McDonnell et al., 2020). Two language models, (1) a conventional unigram LM to inspect the likelihood of every whole word (2) a character 5-g LM to examine sequences of up to five characters, were utilized. Classifiers were built to distinguish each group from the control group, demonstrating a useful signal in each group's language and comparing these classifiers (McDonnell et al., 2020). After that, the correlations between their analytics and classifiers were analyzed to uncover relationships between them and derived insight into quantifiable and relevant mental health signals on Twitter.

Deep neural network (DDN) is another approach that can be utilized for detection of stress as done by Lin et al. (2014), in which the authors presented the analysis of data from four micro-blogs and compared the performance of their proposed four-layered DNN with traditional machine learning algorithms such as Random Forest, SVM, Naïve Bayes. For performance evaluation, they utilized three pooling methods: Max pooling, mean-over-instance, and mean-over-time for each model. Each model performed well or worse, depending on the pooling method. However, the best results were acquired by DNN using mean-over-time pooling.

Neuman et al. (2012) presented another approach “Pedesis” that crawled websites for metaphorical relations in which depression was embedded and extracted the relevant conceptual domains with the NLP method of Dependency Parsing. The domain describes words or phrases that were metaphorical expressions of depression. Human experts further used this information to develop a “depression lexicon” with first- and second-degree synonyms. The lexicon was used to evaluate the level of depression in texts automatically or whether the text is dealing with depression as a topic.

Hidden patterns and high dimension features often help the neural network learn the distinct representation of feature space (Nguyen et al., 2019). The learned features are then used by the trained network to compute the conditional distribution of input vectors. The different architecture of the neural network is being proposed for the domain-specific applications. One of the basic principles is that the architecture is multi-layer perceptron. In this network, each hidden layer takes averaging layers of outputs to compute input from the previous layer and weights. The nonlinear activation function is used at the final/output layer of the network. They update the weights based on the loss function and gradient.

In supervised learning, the network is required to reduce the loss and considered as a nonlinear optimization problem. The weight and bias values are used to optimize the loss. The algorithms mostly fall under the gradient descent technique. The gradient-based techniques start with random points for each input vector. It then several iterations (epochs) are executed for a set of the instance (batches). The trainer computes the loss; it was made by computing the nonlinear objective function for the loss values and gradient. Then, weights are updated in a way that reduces the loss function (Nguyen et al., 2019). The loss is continuously reduced to the convergence point or optimal local minimum. The predictive ability of the neural networks comes from hidden layers and the structure of the architecture. The correct selection of several layers, architecture structure, layers, and hyperparameters helps solve complex problems. The higher-order representation of the input features vector is achieved using the network training (Cho et al., 2014). The learned higher feature representation helps to achieve generalization and increase predictive power. Modern research in the neural network selects the network with low computation complexity and has high prediction power. The number of architecture is proposed over the past two decades (Vinayakumar et al., 2017).

The major difference between architectures is the hidden layers, layers type, shapes, and connection between layers (Sze et al., 2017). Wainberg et al. (2018) introduced the methods for learning higher-dimensional features from the tabular data. The convolutional neural network (CNN) learns features embedding from the image pixels. The pixel data and variation among them increase the learning and predictive power of the network. The translation invariant pixel benefits the network (Wainberg et al., 2018). Many studies were conducted on learning and inference in the visual information processing system that includes wildlife application (Horn et al., 2018), X-ray scans (Rajpurkar et al., 2017), and autonomous driving (Siam et al., 2017). For sequential data, recurrent neural network (RNN) architecture was proposed and used in the natural language process domain, including machine translation, language generation, and time series analysis (Wu et al., 2016; Jouppi et al., 2017; Fawaz, 2020). The RNN model comprises an encoder and decoder framework where the encoder takes the input sequence and decodes it into the vector's fixed length. The model uses different gates to process the input features based on the loss function. The fixed-length vector sometimes loses relevant information (Cho et al., 2014).

Another issue with the RNN encoder and decoder model is the alignment of the input and output vector. Neighbor feature values influence the sequence. Another variant of RNN is the proposal of a new network named as attention mechanism (Cho et al., 2014). It applies the attention method of the input vector by giving certain weights to selected inputs. It makes this selection based on the prioritized importance and position of relevant information after that decoder used the position with context vector and corresponding weights for the higher feature representation. After that mode is then learned the weights to the RNN model for the predictions, the attention weights and context vector learned by using the architecture and feature representation (Lu et al., 2016). Several variations of the network include a soft, hard, and global architecture for the attention mechanism. They proposed the soft attention model (Bahdanau et al., 2015) to help reduce contextual information. The model used the average of the hidden states and then built the context vector. The approach helps to efficiently learn the input feature hidden pattern and reduce the loss.

In hard attention, Xu et al. (2015) computes the context vector from sampling the hidden states. The hard attention reduces the computation cost; however, tuning the architecture is very difficult as the convergence of architecture is difficult. Luong et al. (2015) propose another variation, i.e., local and global attention. Global attention is the intermediate version of soft and hard attention. The model picks the attention point for each input batch. This helps to reach convergence quickly. In the local attention model, they learn the position of the attention vector from the predictive function. The model predicts the attention position. Both local and global attentions are computationally efficient and require to be selected by analyzing the domain-specific data.

## 3. Methodology

**Figure 1:**
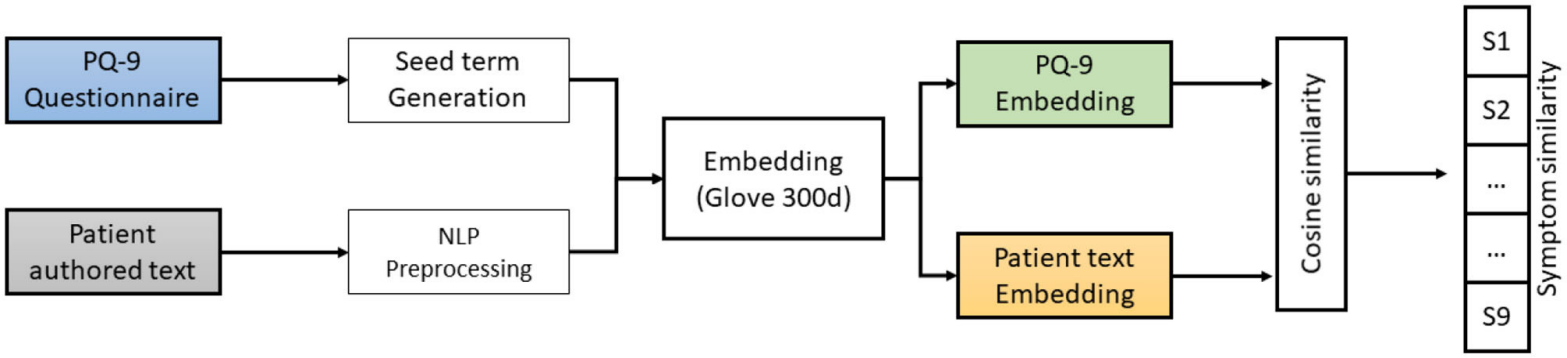
A workflow architecture for estimating PHQ-9 symptoms from the patient-authored texts. PQ, psychometric questionnaire; NLP, natural language processing.

**Figure 2:**
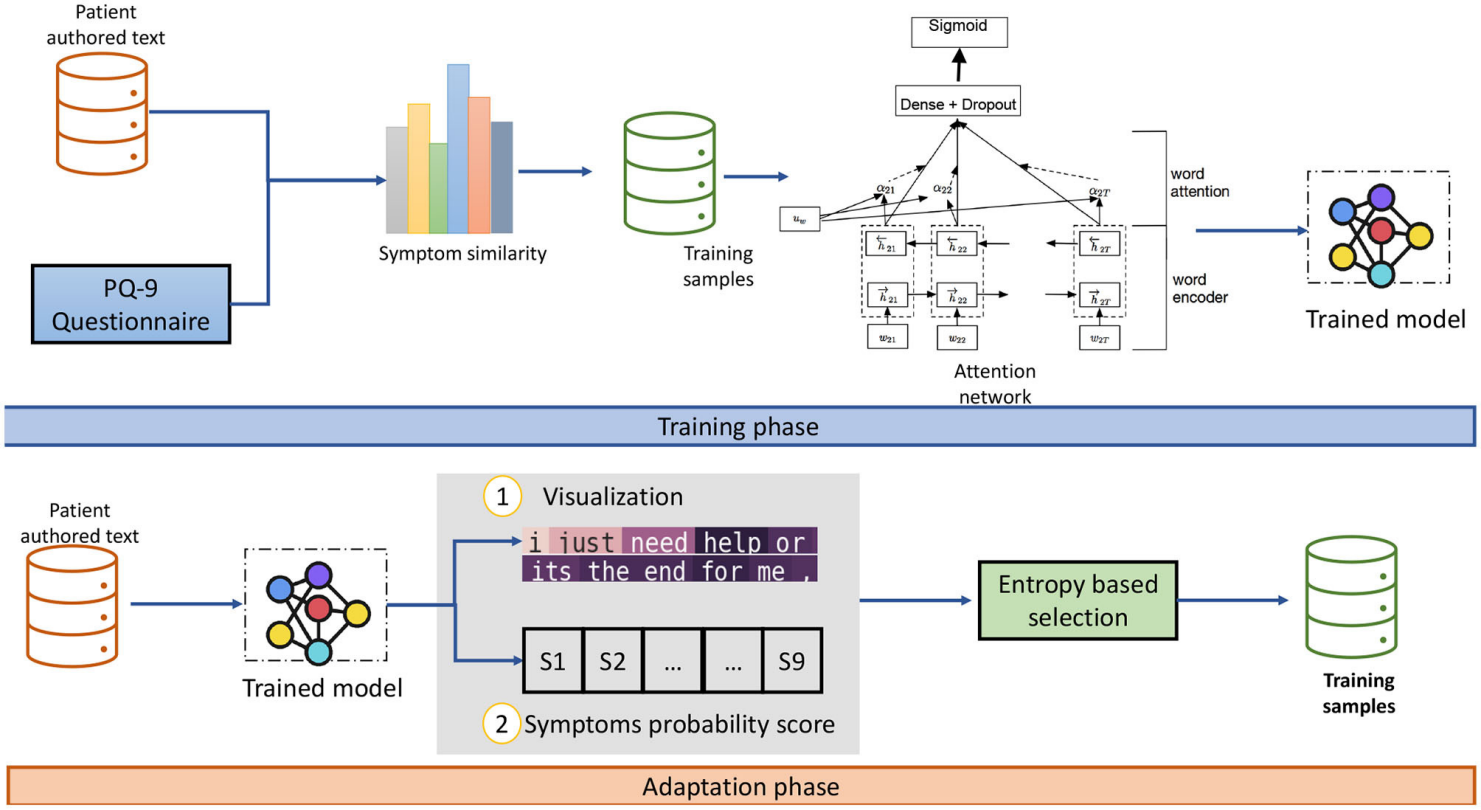
A flow of training and adaption using attention based domain adoption. The visualization and symptoms similarity is suggested for the psychiatrist for note-making and suggesting patient remedy for certain symptoms.

This paper proposes the embedding training method for building a depression symptoms detection model. In this method, as shown in Figures 1, 2, we used the cosine similarity to the PHQ-9 symptoms score. The trained lexical enhanced method is proposed to expand the knowledge and embedding word size for similarity. We explain the proposed method of extracting depression symptoms from the patient's authored text. An example of a patient from the anonymous user is mentioned in the text below.

*I am currently in a pretty bad situation. My depression and anxiety are high, and I can't function or hold down a job or anything like that, so all I do is sit at home all day eating junk food. Each day is extremely boring and hard to get through yet I can't go out into society and function because of my anxiety and depression*.

The diagnosis of mental health issues according to the classification of ICD10 (World Health Organization, 1993) is complicated. The discrepancy of diagnosis is the dynamic nature of symptoms and their degree depending on the patient, treated on a specific disease process at a particular time. Therefore, during the assessment process of mental health issues, the psychiatrists listen to the patient's outlines and extract useful additional information. The psychiatrist's method involves using the standard procedure of questionnaire-based analysis such as PHQ-9 and aided test to assess each assessment's diagnostic reliability according to clinical conditions of the individual with mental health issues. The questionnaire's schemas include symptoms types, their frequency, and summing the frequency to assign the score and then used the score to classify the intensity based on a predefined threshold. For instance, each symptom is represented with nine different questionnaires; those questionnaires' frequency helps classify the behavior into mild, moderate, or severe conditions. The approach is called “Clinical Symptom Elicitation Process” (CSEP) (World Health Organization, 1993). In this research, a major goal is to automate the process through the active learning procedure. Each category of the symptoms is labeled using the patient text's frequency, and overall clinical depression is calculated.

### 3.1. Psychometric Questionnaires (PQ)

There is the number of PHQ-9 for depression and PQ9 is one of the most used questionnaires (Kroenke et al., 2001). The proposed method uses the standard PHQ-9 questionnaire for patient authored text (Kroenke et al., 2001). It is a standard procedure to measure depression symptoms. In standard CSEP procedure, the psychiatrist asks each category's question and observes the patient's response to add the frequency into the class as follows:

- score 0: not at all,
- score 1: several days,
- score 2: more than half the days, and
- score 3: nearly every day.

The PHQ-9 method helps to extract nine distinct behavior types that incorporated DSM-V^2^. These nine symptoms categorize into different disorders such as sleeping, interest, concentration, and food disorder, as mentioned in Table 1 and sample document^3^. After all question-based assessment, the psychiatrist calculates the assessment score. The assessment score indicates the depression level of the patient.

**Table 1:** PHQ-9 questionnaire and seed terms for each symptoms.

**Symptoms**	**PHQ-9**	**Seed terms**
S1	Little interest or pleasure in doing things	Interest
S2	Felling down depressed or hopeless	Feeling, depressed, hopeless
S3	Trouble falling or staying asleep or sleeping too much	Sleep, asleep
S4	Feeling tired or having little energy	Tired, energy
S5	Poor appetite or over eating	Appetite, overeating
S6	Feeling bad about yourself or that you are a failure or have let yourself or your family down	Failure, family
S7	Trouble concentrating on things such as reading the newspaper or watching television	Concentration, reading, watching
S8	Moving or speaking so slowly that other people could have noticed or the opposite being or restless that you have been moving around a lot more than usual	Moving, speaking, restless
S9	Thoughts that you would be better off dead or of hurt yourself	Dead, hurt, suicide

### 3.2. Seed Term Generation

In this research, seed term generation is used for keywords extracted from the PHQ-9 questionnaire. This section describes the generation of the word list of depression symptoms called depression seed term lexicon here. It contains a hand-chosen list of depression symptoms from common resources for psychiatrists, as mentioned in Table 1. Psychologists verified the list of depressive terms since it is critical to base a different synonym list. For each symptom, seed terms are handpicked, then by using Wordnet (Miller et al., 2009), associated hypernyms, hyponyms, and antonyms are extracted. Wordnet is a lexical database for English maintained and developed by Princeton University. Each category of words is maintained in the database, i.e., nouns, verbs, adjectives, and adverbs. Each word in the category possesses different synsets that are used to express unique concepts. The synsets are categorized into semantic and lexicon-based relations. For instance, words having the same synset are synonymous. Empirical analysis found that approximate top 5 terms are beneficial and correlated with original symptom terms. Table 1 seed terms are extended using the Wordnet method. There are various lists of depression symptoms in different classification systems (Mukhiya et al., 2020a). These lists use either clinical or casual symptoms terms depending on whether the poll is a questionnaire to the patient or the clinician. Major classification systems for depression such as DSM-V^4^ and ICD-10 (World Health Organization, 1993) are widely used depression scales that were merged to deduce a fine base list of symptoms (Mukhiya et al., 2020a).

### 3.3. Preprocessing

The preprocessing is an essential part of text processing. Each patient authored text is passed through a different process as follows:

1. Each text is processed and formatted into the UTF-8 encoding scheme. This helps to maintain consistency.
2. Convert each word into lowercase.
3. Remove the tabs or spaces around words.
4. Remove unique characters that do not convey any meaning (#, +, -, *, =, HTTP, HTTPS).
5. Convert text-based words into full words, e.g., *can't* by cannot and so on.

### 3.4. Word Embedding Using Emotional Lexicon

For emotion detection, several methods are proposed in the extensive NLP literature. However, emotional knowledge-based (EKB) systems have not yet been studied. EKB consists of a word sense lexicon and a learned diverse contextual embedding. We propose the embedding that takes contextually diverse words by combining the depression lexicon (based on word sense) and emotional knowledge from online forums. Emotional knowledge consists of words that represent context and feelings. For each word token in the patient text, we extracted the word embedding using a 300 dimension pre-trained model for global vector for word representation (Glove) (Pennington et al., 2014). The Glove-based vector embedding is used to project the context in vector space. The embedding represents the learned sentence structure. The extracted embedding helps to captivate the semantic composition of the text. Each word vector is distributed based on the hypothesis that *"You shall know a word by the company it keeps"* (Charles, 2000). The *co-occurrence frequencies* of the vectorized words is calculated based on the linguistic patterns. The learned model is produced from the author's unique word and represented with a fixed-length vector. A similar word is located nearby. Most of the pre-trained embedding is for general-purpose communication. Therefore, a *pre-trained model* does not apply to emotional analysis. We extend the corpus by using the word sense model and transfer learning method of training the custom mental health model. The reason for this is that most of the embedding is trained on open-source data, i.e., *(Wikipedia texts)* and sentiment knowledge *(Twitter data)*. The word *sad* and *happy* convey the meaning of *feelings*. However, these words represent a different mental state. Therefore, it is needed to extend the embedding using word sense.

The emotional lexicon based on the word sense helps to show promising results. The fine grain classification can be achieved by using custom embedding for the classification of various symptoms. Part of speech tagging is used and extracted the words that contain the (*noun, verb, adverb*, and *adjective*). We used the corpus *D* consist of the set of texts, *D* = {*t*_1_, *t*_2_, …, *t*_*n*_} and the *WordNet* is used to extract synonyms, antonyms, hypernyms, and physical meaning for each extracted part of speech. As a results, we get the emotion words *W* = {*w*_1_, *w*_2_, …, *w*_*K*_} for each documents. The emotion represents a domain-specific contextual corpus. After that, vocabulary is built using the *W* set used to train the model. The resultant embedding is learned vector *V*, i.e., $V=\left\{v_{1},\;v_{2},\ldots ,v_{m}\right\}\in {\mathbb{R}}^{m\times \delta }$ where *δ* is the word vector dimension. The sentence embedding is obtained by averaging each word vector in the patient author text. The vector represents word sense and emotional knowledge. The trained model is used to convert the patient author text into a vector and all nine symptoms from the PHQ-9 questionnaire lexicons. The corresponding two embeddings are passed to the cosine similarity method. For every nine symptoms, we have a similarity value ranging between 0*and*1. Given two vectors, vector *X*, which is the patient author text, and vector *Y*, representing the symptoms lexicon, we use *V* to create textual features into semantically aware vectors. The similarity between two embeddings represents that authored text is closely related to certain symptoms, as shown in Figure 2.

### 3.5. Dataset

The dataset is gathered from an online forum, website, and social media site (Mukhiya et al., 2020a). Amazon Mechanical Turk^5^ service is used to label the 500 texts (Mukhiya et al., 2020a). The remaining data are annotated by using the proposed embedding method. The labeling is done using the PHQ-9 rating method, i.e., such that 0 indicates not depressed, 1 mildly depressed, 2 moderately depressed, and 3 severely depressed (Mukhiya et al., 2020a). We convert the annotation into a binary class for each symptom, where 0 indicates the absence of symptoms and 1 indicates the presence of symptoms. The gathered data are shown in Table 2.

**Table 2:** The statistical summary of the training and testing set.

**Type**	**Statistics**
Corpus size (Number of posts collected)	15,044
Number of sentences	133,524
Average sentences per post	8.87
Average words per post	232
Training set size (Number of posts)	14,944
Testing set size (Number of posts)	100

### 3.6. Deep Learning Model

As a **baseline**, we used a feedforward neural network. The Glove embedding is used to extract all the tokens from the text. The averaging method is used to average the comment length to a uniform size. The model consists of hidden layers (30, 20, 10) with a ReLU activation function (Nair and Hinton, 2010). Table 3 shows that our goal is multi-label classification (of nine distinct symptoms). The last layer contains the sigmoid function with nine units. The cross-entropy function is used as the loss function. The model is defined as follows:

$$\begin{array}{c} h_{1}=\mathrm{ReLu}\left(xW_{1}+b_{1}\right)\\ h_{2}=\mathrm{ReLu}\left(h_{1}W_{2}+b_{2}\right)\\ h_{3}=\mathrm{ReLu}\left(h_{2}W_{3}+b_{3}\right)\\ \;\hat{y}=\sigma \left(h_{3}W_{4}+b_{4}\right)\\ \;J=CE\left(y,\hat{y}\right)=-\overset{6}{\underset{i=1}{\sum }}y_{i}\log\left({\hat{y}}_{i}\right) \end{array}$$

where

$$\begin{array}{l} x\in {\mathbb{R}}^{B\times 300},h_{1}\in {\mathbb{R}}^{B\times 30},h_{2}\in {\mathbb{R}}^{B\times 20},h_{3}\in {\mathbb{R}}^{B\times 10},\\ \hat{y}\in {\mathbb{R}}^{B\times 6},y\in {\mathbb{R}}^{B\times 6} \end{array}$$

An ROC curve is used, true positive rate [*TPR* = *TP*/(*TP* + *FN*)], and false-positive rate (*FPR* = *FP*/(*FP* + *TN*) as performance metrics.

**Table 3:** A snippets of dataset used.

**Text**	**S1**	**S2**	**S3**	**S4**	**S5**	**S6**	**S7**	**S8**	**S9**
It is too much to handle. The depression and anxiety. Tried so many ways to get better including varying cocktails of meds but I feel so hopeless. Last semester and I think I'm going to fail.	0	1	0	0	0	0	0	0	0
Having a very bad day today. Haven't even got dressed yet might not bother at all today. Don't really know why I keep going. Feel so very very sad and……	0	0	1	0	0	0	0	0	1
Hi all I'm after a bit of advice. I think my partner is depressed and I told him he needs to go to the doctors. He works away Monday to Friday and is stressed out at work working as a lorry driver he does long hours (70+ a week)…	0	1	0	0	0	0	1	0	0

RNN with GRU is used, and LSTM cells as the RNN architecture performed well for the sequential task. The LSTM network allows for long-distance information preservation. In LSTM unidirectional architecture, a final time step of the hidden state can be fed into the output layer. We found that the element-wise average method overall timesteps' hidden state performed better for the input to the final layer of our architecture during empirical analysis. We also used the bidirectional LSTM architecture that read input token lists starting at the end and set one parameter for forward unrolled LSTM. Therefore, each token position has two input states that concatenate to form the output state, extending for the attention layer. The dropout ratio of 0:5 is set to avoid overfitting and regulation of the LSTM layer.

$$\begin{array}{l} i_{t}=\sigma \left(x_{t}W^{\left(i\right)}+h_{t-1}U^{\left(i\right)}\right)\\ f_{t}=\sigma \left(x_{t}W^{\left(f\right)}+h_{t-1}U^{\left(f\right)}\right)\\ o_{t}=\sigma \left(x_{t}W^{\left(o\right)}+h_{t-1}U^{\left(o\right)}\right)\\ {\tilde{c}}_{t}=\tanh\left(x_{t}W^{\left(o\right)}+h_{t-1}U^{\left(o\right)}\right)\\ c_{t}=f_{t}\circ c_{t-1}+i_{t}\circ {\tilde{c}}_{t}\\ h_{t}=o_{t}\circ \tanh\left(c_{t}\right) \end{array}$$

The attention method is proposed to utilize word importance in text (Yang et al., 2016). We added the attention method in addition to the LSTM layer. This addition helps to extract informative words for the classification task. The attention output vector is fed as the input to the dropout layer. The formal representation of the network is mentioned below. Traditionally, supervised learning required a large labeled dataset for the training of large networks. The label data are the main requirement and dependency of the application. The active learning model is the process to generate the relevant set of data that have the highest predictive significance to training a supervised model. The active learning model is used in applications where the amount of data is too large to do manual labeling. In this research, we used the similarity-based features to label a small set of data smartly and after that using the entropy-based instance selection method of train on the full dataset. The entropy-based instance selection mechanism (Holub et al., 2008) is adopted to expand the low number of instances and chose the data distribution. This process helps to expand knowledge with time.

$$\begin{array}{c} v_{t}=\tanh\left(h_{t}W_{a}+b_{a}\right)\\ s_{t}=v_{t}u_{a}^{⊤}\\ \alpha_{t}=\frac{\exp\left(s_{t}\right)}{\sum_{t=1}^{T}\exp\left(s_{t}\right)}\\ \tilde{h}=\overset{T}{\underset{t=1}{\sum }}\alpha_{t}h_{t} \end{array}$$

## 4. Experimental Result and Analysis

First, the patient-authored text is converted into the emotional-based lexicon and trained on neural networks for experimentation. We used a 300 dimension Glove vector for vectorization. The embedding is used to convert a text and a nine symptom lexicon into a vector. Then, two vectors are used to find the similarity based on the cosine similarity. The similarity is used to label the text. The label text is then further trained on a different architecture. Next, the different architecture is evaluated based on an ROC curve, precision, and recall. For each architecture, we used Adam optimizer (Kingma and Ba, 2014), hyperparameter tuning is done by keeping the learning rate static to 0.0005, which in turn helps to reduce the training loss. Table 4 shows the architecture performance, and the attention method helps achieve the highest ROC on the test set. For each architecture, we changed the cell type as well as the hidden size. In addition to the LSTM directional layer, we added the attention method to improve model performance.

**Table 4:** The mean ROC–Area Under the ROC Curve values of training and testing set.

**Architectures**	**Train**	**Test**
Baseline	0.89	0.81
LSTM	0.65	0.38
Bidirectional LSTM	0.91	0.8
Bidirectional_LSTM_Attention	**0.91**	**0.85**

*The bold value represents the highest ROC-AUC value*.

Simultaneously, other models tend to overfit as they performed well on the training set but did not perform well on the development and testing set. Three steps were followed to prevent overfitting. First, the model was run for a longer time (1000, epochs). Second, the concept of early stopping methods was used to save the model progressively. Third, a gradient clipping method was used to ensure and avoid gradient issues (Chen X. et al., 2020).

**Figure 3:**
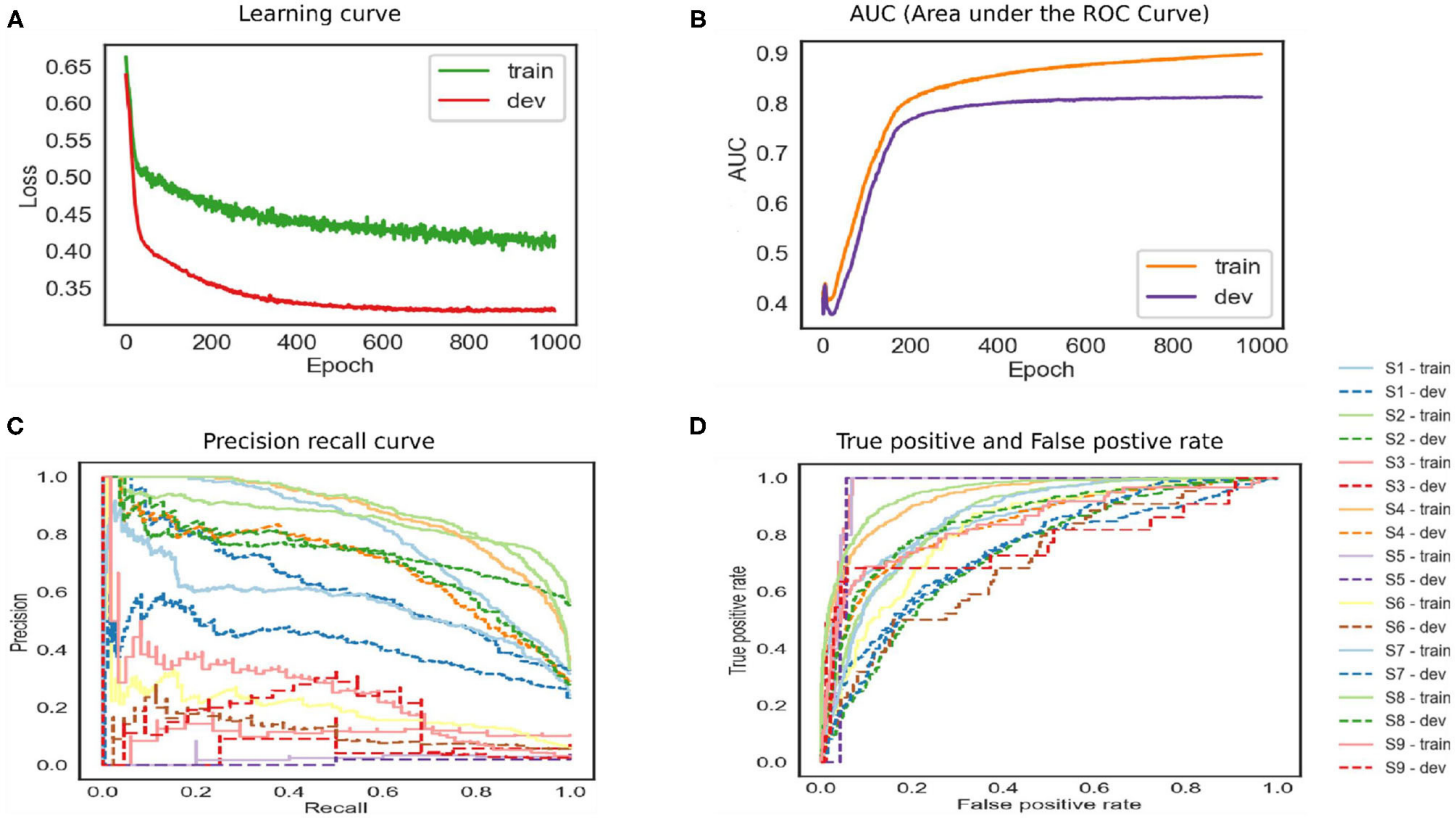
The feed-forward neural network baseline model.

The baseline model performance is shown in Figure 3. The training loss reached 0.45, and the testing loss is 0.33. The training ROC is 0.89, and the development set is 0.81. The model tends to overfit and has close to the upper left corner—the precision-recall curve under the different threshold value is relatively low false-positive rate. The model did not perform well. Therefore, the architecture is not optimal for the given data. The depression data depend upon the sequence of words that were not preserved by a simple network. In other words, an architecture that favors sequences and stores important word information is most likely required for stronger results.

**Figure 4:**
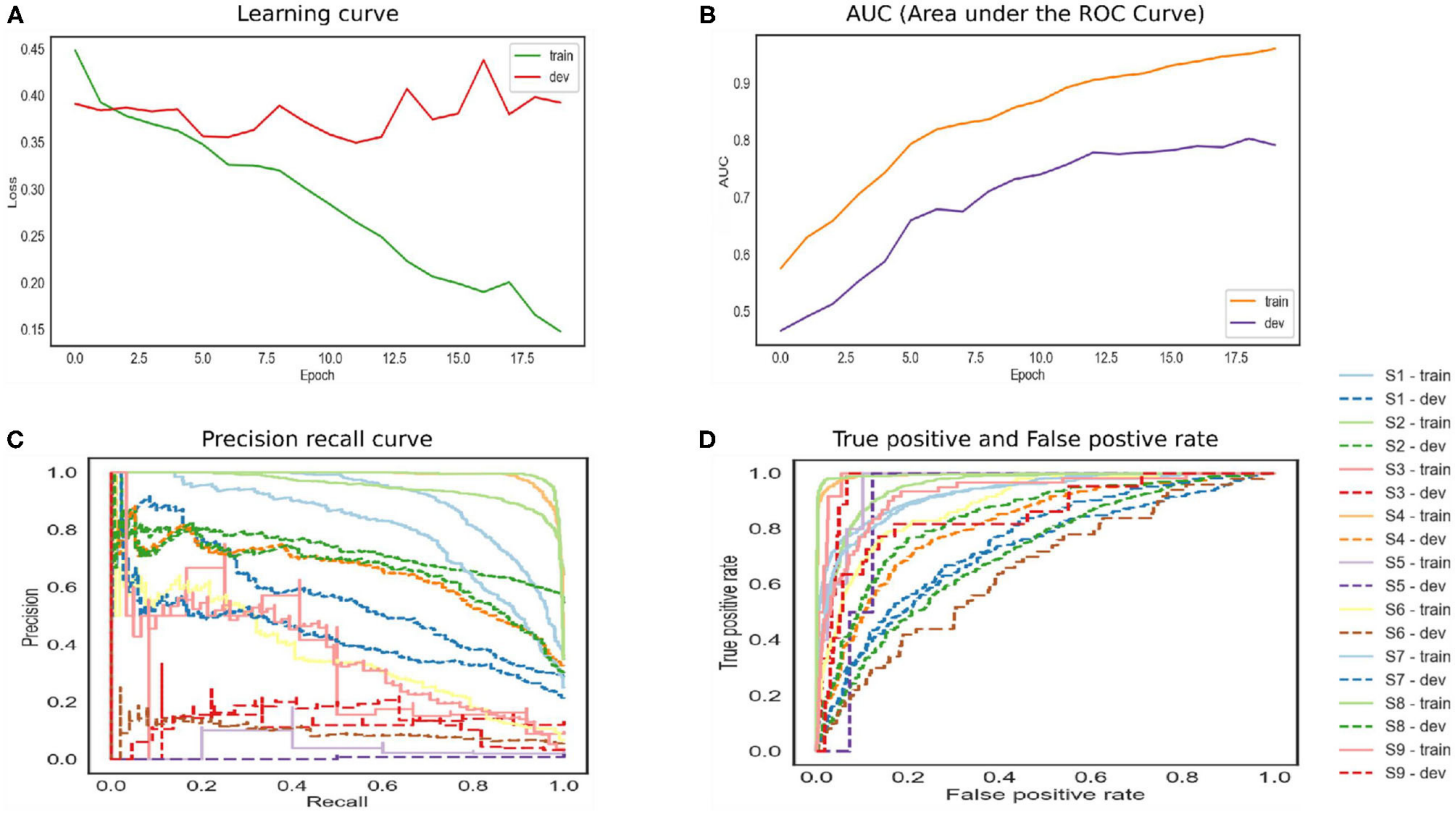
The performance of Long Short-Term Memory (LSTM) model.

The LSTM network is shown in Figure 4. The training and developing loss is optimal as we set epochs as 20. However, the model achieved ROC 0.79. The precision–recall curve indicates that the model is not able to learn effectively. This model also does not perform well on this dataset. The model has to remove gradient issues as the cell has to move data from one cell to another. The cell becomes complex due to the computational cost of the gates. Moreover, the LSTM architecture required more fine tuning and training for a longer period. For real-time applications, the network should store information for a longer time to achieve human-level performance. For instance, human habits of dividing sources of information into small chunks for ease of remembering past events. Likewise for feedforward networks, LSTM also favors small weight initialization. In summary, LSTM behaves almost the same as a feedforward network.

**Figure 5:**
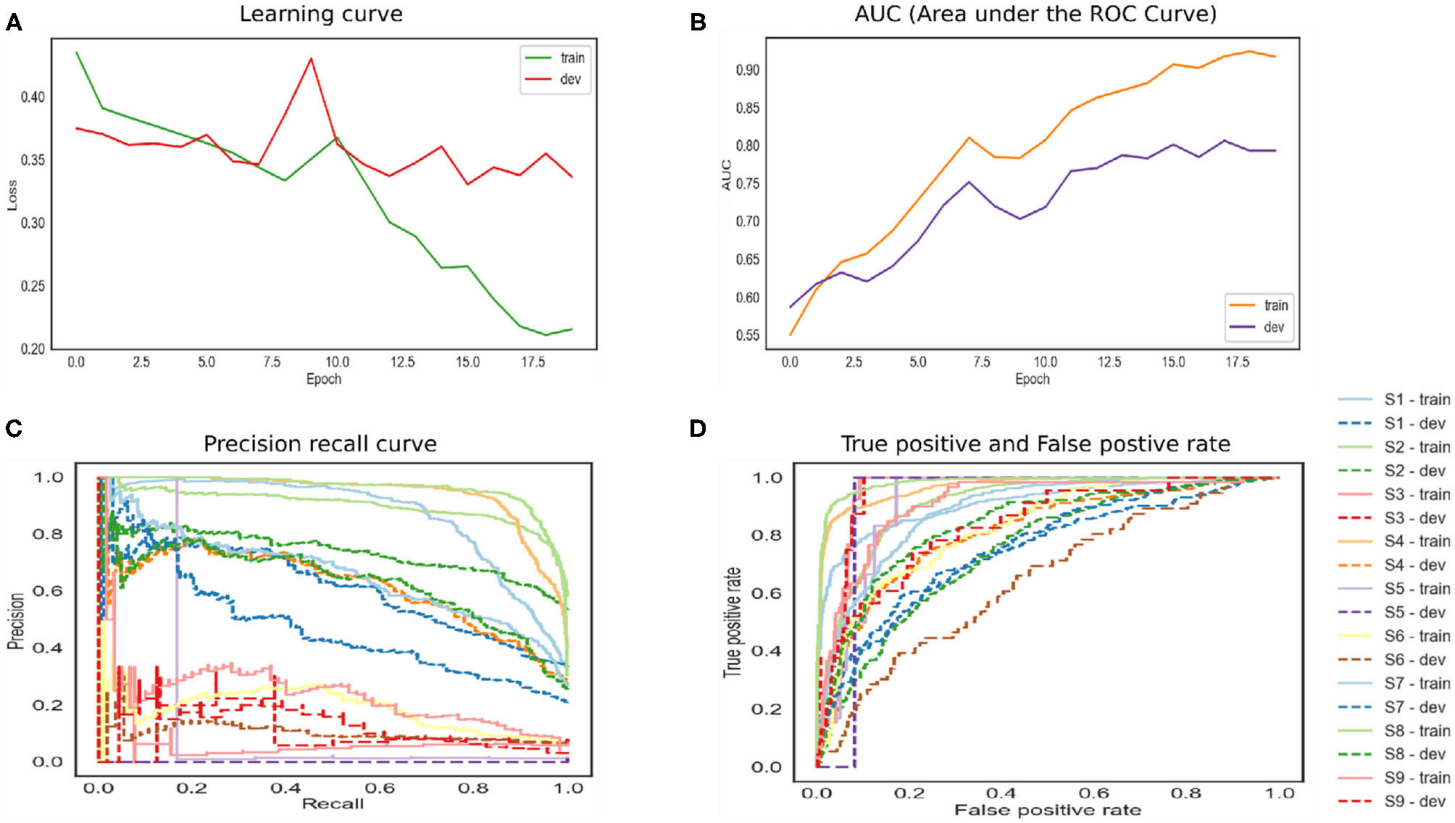
The performance of bidirectional Long Short-Term Memory (LSTM) model.

The bidirectional model achieved high accuracy as shown in Figure 5. The reason for this is that the model runs in two directions from past to future and vice versa. The two hidden state models preserve the information from the future as well as the past. The two independent RNNs are parallelly performed that allow the networks to have backward and forward connections. The trained and development set has the lowest error. The precision–recall curve in the top corner represents high recall and high precision, which depicted the low false-positive and false-negative rates. The BILSTM model takes each hidden state, which depended upon the previous state. This creates a huge problem as the network is required to wait for data dependency. The long-range dependencies affect the performance as it is a challenge to memorize the information for a long time.

**Figure 6:**
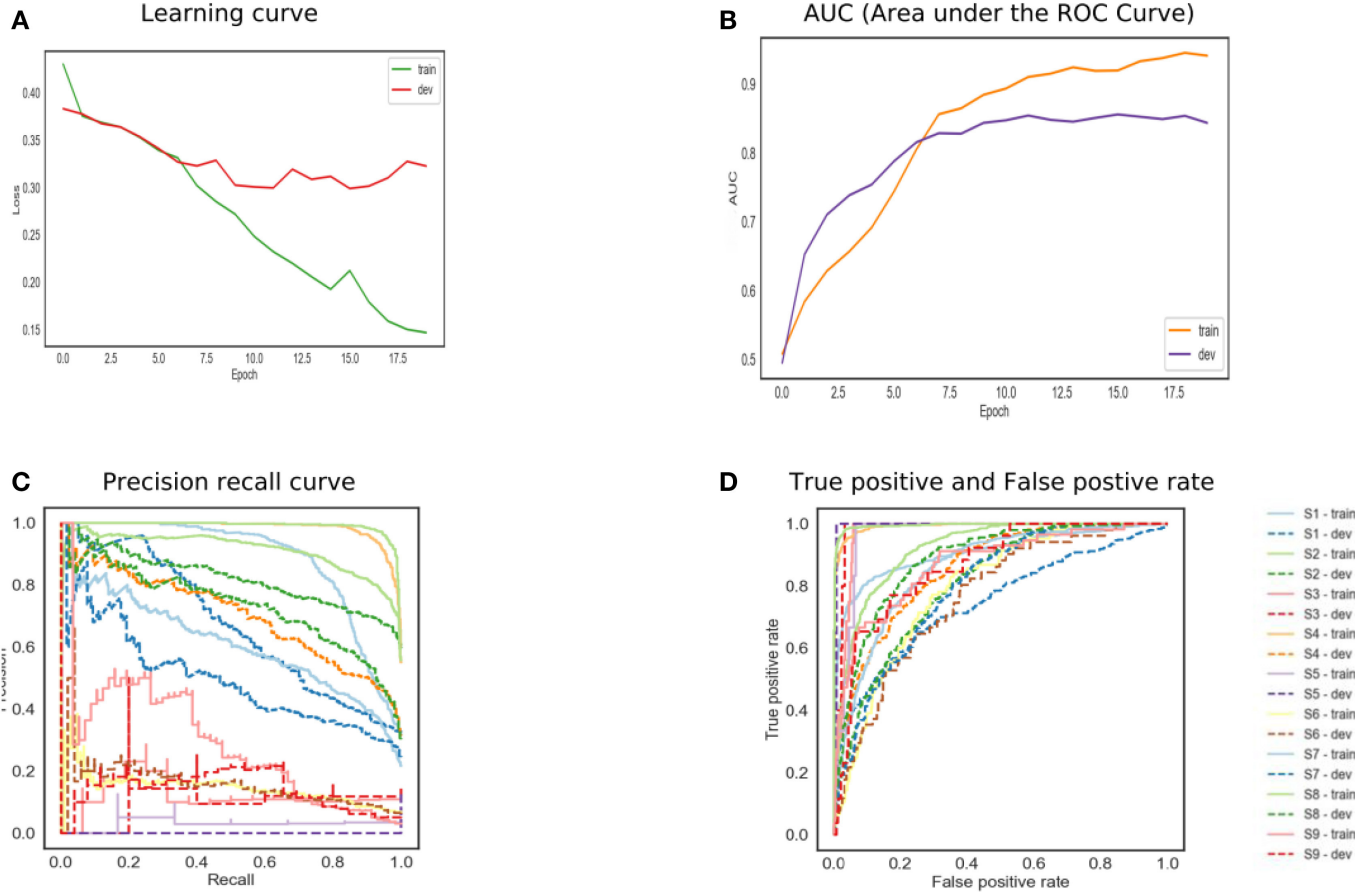
The performance of bidirectional Long Short-Term Memory (LSTM) with attention.

In Figure 6, the attention mechanism is used with a bidirectional LSTM. The model normalized attention weights to selected high-quality words that understood and correlated with the classifier. The model can have inadequate training and development set error. The training model achieved 0.91 ROC, and the development set is 0.85. The high-performance results in a high true positive rate. The results support the existence of essential words that help to classify the depression symptoms. The network also helps to reduce the computation cost by focusing on certain words. The reason is that the model can recognize the target word in the task, and it learned the subject's meaning in both directions. Due to the complex nature of the mental health data, a large number of vocabulary and grammatical permutations can increase the performance.

**Figure 7:**
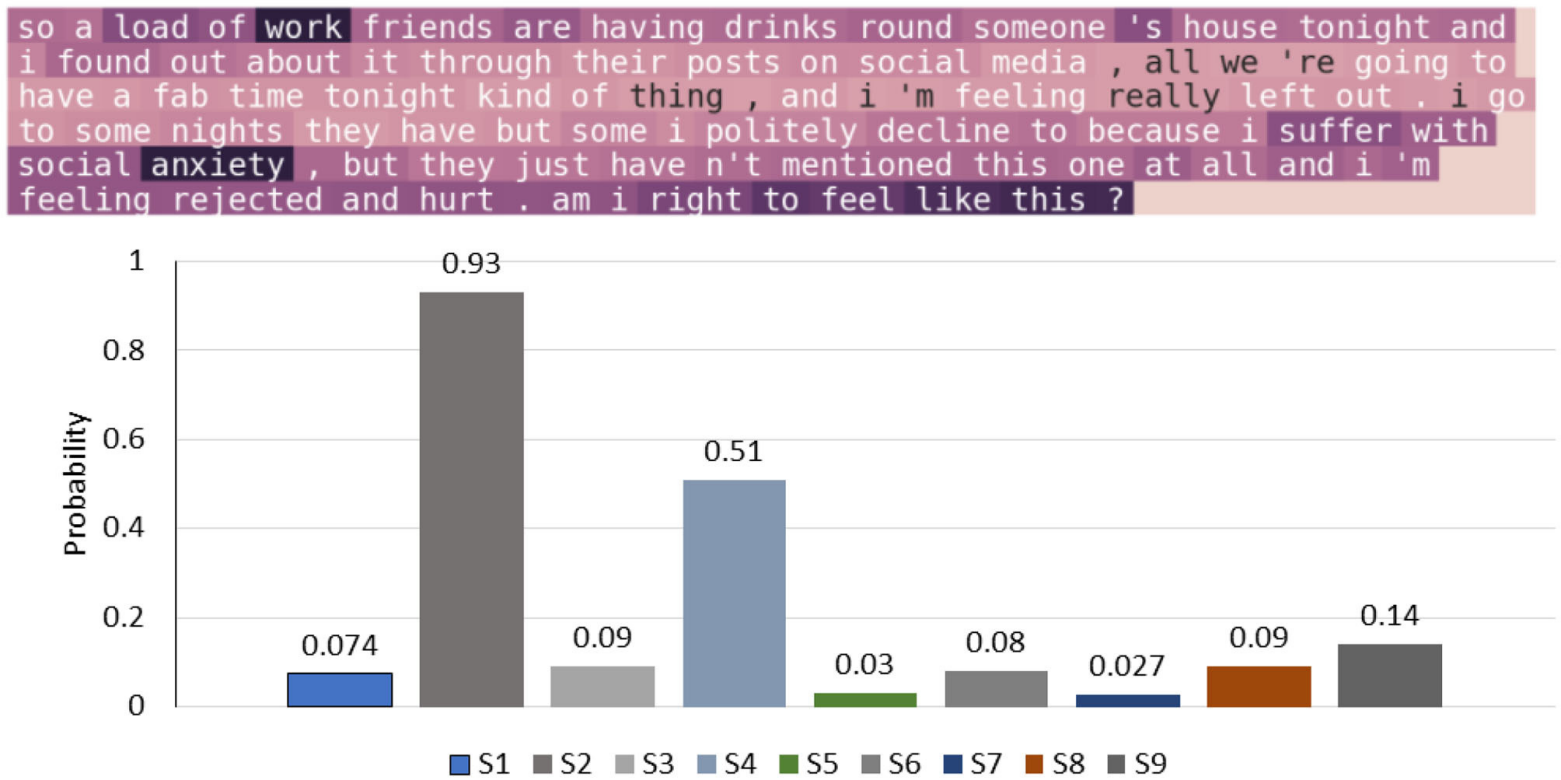
An example patient-authored text and visualization of depression symptoms extracted by our approach.

In Figure 7, the attention method is used to compute the normalized attention weights for every word in a sentence of a patient authored text; the visualization is used to help the psychiatrist to see the trigger points. The weights of qualitative words are highlighted, which represent *work, anxiety, feel, and suffer*; they indicate two symptoms, i.e., S1 (felling down depressed or hopeless) and S4 (feeling tired or having little energy). The model also successfully highlights the critical points and the relevant words for the symptoms that is helpful for classifications tasks.

## 5. Conclusion

The applications of NLP and deep learning for clinical text analysis have greatly improved in recent years. In the past studies, Patient's authored text data are used to extract symptoms, and a limited number of studies have been conducted to extract mental health symptoms. Moreover, adoption methods using mental health has also not been well discussed in related works. This paper presents a semi-supervised learning method for labeling and training an active learning model. The active learning model is able to expand its knowledge with timestamp. Through our symptom-based visualization system, as well as the symptoms themselves, psychiatrists is able to make and recommend relevant programs for adequate therapy effectively. In the designed system, IDPT helps with computerized exercises for psycho-education, and NLP helps to provide an elegant way to adapt and offer proper visualization. The LSTM and attention model help to achieve high accuracy for the prediction of symptoms. The bidirectional LSTM with an output attention layer was successfully able to perform multi-label classification for symptoms. The active learning model was able to expand knowledge with time. Our model achieved 0.85 ROC, helped to visualize the attention-based words, and recommended the suggested symptoms. The proposed method performs adaptation in IDPT systems that automatically learns from patient's authored texts for psycho-education exercises. Through our results, the adapted intervention provides personalized feedback on recommended exercises. In the future, we will try to embed a character-level text classifier, as well as stronger regulations that may be able to increase the performance of our model and reduce overfitting issue.

## Data Availability Statement

The data analyzed in this study is subject to the following licenses/restrictions: Dataset is available upon request. Requests to access these datasets should be directed to Suresh Kumar Mukhiya, Suresh.Kumar.Mukhiya@hvl.no.

## Author Contributions

UA and JL investigated the main idea and wrote the draft of the manuscript. GS revised and proofread the manuscript. SM and YL helped for the evaluation part of the experiments. All authors contributed to the article and approved the submitted version.

## Conflict of Interest

The authors declare that the research was conducted in the absence of any commercial or financial relationships that could be construed as a potential conflict of interest.

## Footnotes

^1^https://www.who.int/news/item/05-10-2020-covid-19-disrupting-mental-health-services-in-most-countries-who-survey.

^2^https://www.psychiatry.org/psychiatrists/practice/dsm.

^3^https://www.uspreventiveservicestaskforce.org/Home/GetFileByID/218.

^4^https://www.psychiatry.org/psychiatrists/practice/dsm.

^5^https://www.mturk.com/.
